# Highly efficient soft x-ray spectrometer for transient absorption spectroscopy with broadband table-top high harmonic sources

**DOI:** 10.1063/4.0000096

**Published:** 2021-06-30

**Authors:** Carlo Kleine, Maria Ekimova, Marc-Oliver Winghart, Sebastian Eckert, Oliver Reichel, Heike Löchel, Jürgen Probst, Christoph Braig, Christian Seifert, Alexei Erko, Andrey Sokolov, Marc J. J. Vrakking, Erik T. J. Nibbering, Arnaud Rouzée

**Affiliations:** 1 Max Born Institute for Nonlinear Optics and Short Pulse Spectroscopy, Max Born Str. 2a, 12489 Berlin, Germany; 2 Nano Optics Berlin GmbH, Krumme Strasse 64, 10627 Berlin, Germany; 3 Institute of Applied Photonics (IAP) e.V., Rudower Chaussee 29/31, 12489 Berlin, Germany; 4 Helmholtz-Zentrum Berlin für Materialien und Energie GmbH, Albert-Einstein Strasse 15, 12489 Berlin, Germany

## Abstract

We present a novel soft x-ray spectrometer for ultrafast absorption spectroscopy utilizing table-top femtosecond high-order harmonic sources. Where most commercially available spectrometers rely on spherical variable line space gratings with a typical efficiency on the order of 3% in the first diffractive order, this spectrometer, based on a Hettrick–Underwood design, includes a reflective zone plate as a dispersive element. An improved efficiency of 12% at the N K-edge is achieved, accompanied by a resolving power of 890. The high performance of the soft x-ray spectrometer is further demonstrated by comparing nitrogen K-edge absorption spectra from calcium nitrate in aqueous solution obtained with our high-order harmonic source to previous measurements performed at the electron storage ring facility BESSY II.

The recent development of broadband table-top extreme ultraviolet (XUV) and soft x-ray sources based on high-order harmonic generation (HHG) has led to a revolution in our capability to investigate the physical and chemical properties of matter under non-equilibrium conditions. With pulse durations reaching the attosecond time scale^1,2^ and photon energies extending into and above the water window spectral range,^3–10^ these sources enable the investigation of ultrafast electronic dynamics, including strongly coupled electron-nuclear dynamics in photoexcited molecules^11–13^ and materials,^14^ with exquisite spatiotemporal resolution. In particular, ultrafast x-ray absorption spectroscopy (XAS) at atom-specific absorption edges has become a powerful tool to investigate both the local transient electronic and structural environment of molecules and solid-state matter.

Initially, ultrafast XAS studies were limited to the extreme ultraviolet (XUV) spectral range by conventional high-order harmonic sources pumped with 800-nm Ti:Sa lasers. Femtosecond and attosecond XUV transient absorption spectroscopy have been extensively used in gas-phase samples in order to investigate ultrafast valence electronic motion,^15^ light-induced coupling between states,^16^ and ultrafast bond-breaking in iodine compounds.^17^ In the condensed phase, among other things, carrier-induced bandgap reduction and carrier–carrier interaction dynamics in the conduction band of silicon^18^ and femtometer-scale phonon dynamics in polycrystalline LiBH_4_^19^ have been investigated by time-resolved XUV absorption spectroscopy. Ultrashort soft x-ray pulses in the water window spectral range have been available following the development of high-order harmonic sources driven by long-wavelength, intense infrared lasers, with wavelengths above 1.6 *μ*m.^3–6,9,10^ These sources, coupled with XAS, have already permitted studies of the ultrafast structural dynamics of photoinduced ring-opening reactions in cyclohexadiene^20^ and furfural,^21^ ultrafast intersystem crossing in acetylacetone^22^ and strong-field induced dissociative ionization in CF_4_.^23^ More recently, the electronic, vibrational, and rotational molecular dynamics during and following strong field ionization of nitric oxide have been studied by time-resolved XAS at the N K-edge near 400 eV^24^ and attosecond charge carrier dynamics in TiS_2_ semi-metallic materials have been characterized by attosecond transient absorption spectroscopy at the Ti L-edge near 460 eV.^25^ First, steady-state and time-resolved XAS of molecules in solution have also been demonstrated^10,26^ using table-top soft x-ray sources.

Despite the overall progress achieved in ultrashort soft x-ray pulse generation, the decrease in efficiency of the HHG process upon an increase in pump driving wavelength represents a major challenge to the further development of ultrafast XAS at high photon energies. Typical HHG photon fluxes of 10^4^ to 10^5^ photons/s/eV at the N K-edge are reached with an HHG source.^4,9^ Given the losses within a typical HHG beamline and the finite detection efficiency, the number of detected photons in a HHG-based XAS experiment further decreases to less than 100 photons/s/eV.^24^ Assuming a shot noise-limited photon detection, a minimum acquisition time of 100 s is therefore required to record a high harmonic spectrum near the N K-edge with an uncertainty of 1%. X-ray spectrometers with high-throughput and high-detection efficiency that would allow to decrease this relatively long acquisition time are mandatory for recording multiple absorption spectra in pump-probe studies with sufficient signal-to-noise ratio, considering that the differential pump-induced absorption is a small fraction of the actual XUV absorption.

To maximize the transmitted flux, most XAS experiments with table-top HHG sources rely on a compact flat-field grazing incidence spectrometer equipped with a commercially available aberration-corrected concave variable line-space (VLS) grating, which combines the dispersion and focusing of the incoming radiation in a single optical element. In addition to that, a VLS grating provides a flat focal plane of the spectral image over a wide wavelength range and is therefore particularly adapted to recording of the broadband HHG spectrum on position-sensitive detectors such as microchannel plates and CCD sensors. However, the diffraction efficiency of commercially available VLS gratings is generally quite low in the water window spectral range (typically below 3%). As an alternative, new soft x-ray spectrometers have recently emerged that make use of high-efficiency reflective zone plates (RZP).^27–30^ For example, a multi-channel RZP monochromator for ultrashort soft x-ray pulses has recently been constructed^31^ and commissioned at the FemtoSpeX beamline at BESSY II,^32^ showing an impressive throughput of 21% in a photon energy range between 410 and 1333 eV, accompanied by an energy resolving power of E/ΔE=500.

Here, we describe the design and performance of a new soft x-ray spectrometer for XAS with table-top high-order harmonic sources. The instrument is based on the Hettrick–Underwood optical scheme^33^ and consists of a grazing incidence concave cylindrical mirror for one dimensional focusing and a high transmission efficiency RZP with an average line density of 700 l/mm for dispersion. We demonstrate a resolving power of 890 at a central photon energy of 410 eV and a RZP transmission efficiency of 12%. The substantially improved performance in transmission efficiency and resolution is illustrated with solution phase measurements at the calcium L_2_, L_3_-, and nitrogen K-edges.

The design of the spectrometer has been adapted to a high-order harmonic source, developed recently at the Max Born Institute (see Ref. 10 for details). Briefly, the soft x-ray beam obtained by HHG is propagated under vacuum conditions and is focused onto a sample by a 4° grazing incidence Ni-coated toroidal mirror (see M1 in Fig. 1). The sagittal and tangential radii of curvature of the toroidal mirror are set to 41.8 and 8601.4 mm, respectively, leading to a 30 cm focal distance in both directions. In this configuration, a (1:1) image of the HHG source is provided 60 cm after the toroidal mirror. This point corresponds to the position where the sample is ordinarily placed and serves as source point of the spectrometer unit. The source size and divergence of the beam are estimated around 50 *μ*m FWHM and ≈0.5 mrad, respectively.

**FIG. 1.**
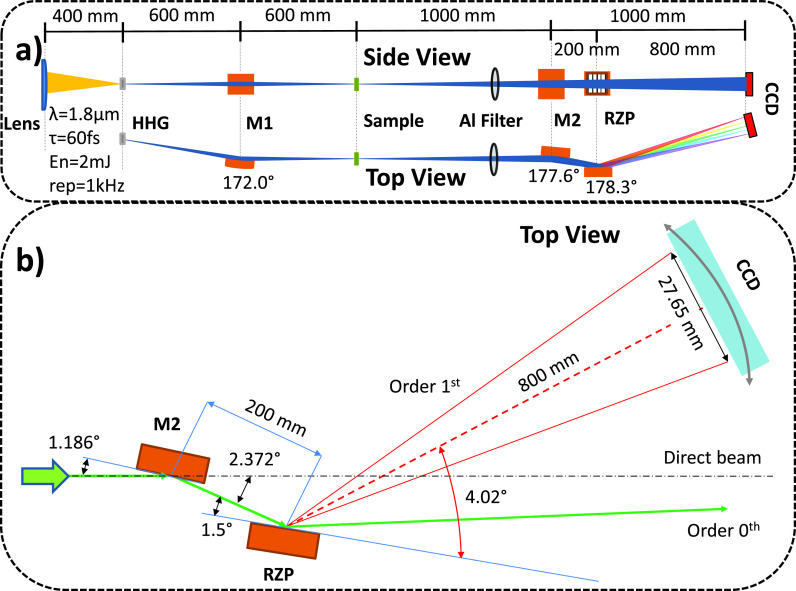
Schematic of the experimental setup. (a) Top and side views of the soft x-ray experimental setup, including the HHG source, the sample, and the soft x-ray spectrometer. (b) Optical design of the RZP-based soft x-ray spectrometer. In this configuration, the CCD detector covers the spectral range from 250 eV to 600 eV.

The spectrometer has been designed to enable a resolving power of at least 600 in a photon energy range between 200 eV and 600 eV. Due to the challenge in producing a perfect aberration-corrected grating device on spherical or toroidal surfaces, a spectrometer according to the Hettrick–Underwood design was chosen that permits the use of a planar RZP (see Fig. 1). The spectrometer consists of a 100 × 30 × 8 mm^3^ Ni-coated concave cylindrical mirror (M2) with 48.3 m radius of curvature, an RZP fabricated on a 100 × 30 × 10 mm^3^ planar single crystal silicon substrate, and a two-dimensional CCD detector (Greateyes GE 2048 512 BI UV1). The size of the concave mirror and the RZP substrate was chosen to accommodate an acceptance angle of 2 mrad in the meridional (top view) and 3.5 mrad in the sagittal (side view) plane, respectively. The 1.186° grazing incidence concave mirror M2 has a reflectivity above 90% and allows focusing of the HHG beam on the CCD detector, while the RZP is used as dispersive element. The RZP follows the Fresnel zone condition providing theoretically zero aberration for a central photon energy of 410 eV. It has an average line density of 700 l/mm, an angle of diffraction of 4.02° at 410 eV, and a grazing angle of 1.5°. Its design parameters are based on simulations using RAY-UI,^34^ a ray tracing program and design tool for synchrotron radiation beamlines, and the successor of the well-known BESSY ray tracing code RAY-UI developed since 1984.^35^

The calculated energy resolving power of the spectrometer as a function of the source size and the distance between the RZP and the CCD detector is shown in Fig. 2. An increase in the source size from 40 to 80 *μ*m (FWHM) results in a large decrease in the resolving power from 950 to 700 at a photon energy of 410 eV. Moreover, the resolution of the spectrometer is influenced by displacement of the CCD detector around the focal plane. In order to achieve the highest energy resolving power, the mirror M2 and the RZP are mounted on motorized ultrahigh vacuum compatible hexapod nanopositioning systems (SmarAct model SMARPOD 70.42), placed 1.0 and 1.2 m away from the source point (i.e., sample), respectively. Furthermore, the CCD detector is mounted on a circular railway that allows rotation of the camera position around the RZP in a range from 0°, that is, normal incidence, to a maximum angle of 5.5°. At normal incidence, the soft x-ray beam can be recorded directly with the CCD detector by moving the concave mirror and RZP out of the beam path and blocking the 1.8 *μ*m driving pulse and the low-order harmonics with a combination of thin metal filters (e.g., 200-nm Al filter and 50-nm Ti filter). Thereby, the efficiency of the HHG process and the divergence of the soft x-ray beam can be directly monitored and optimized.

**FIG. 2.**
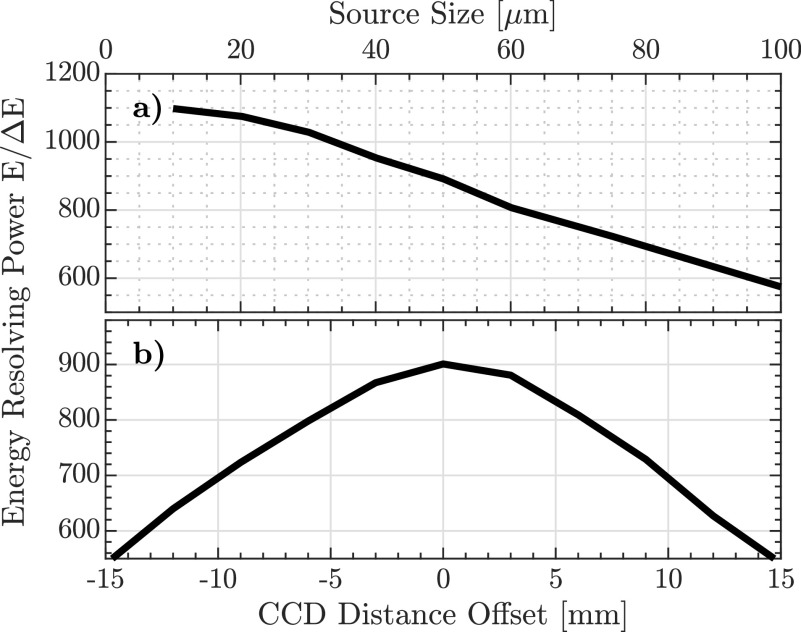
Calculated energy resolving power of the RZP spectrometer at 410 eV, (a) as a function of the soft x-ray focal spot size (FWHM) and (b) a distance offset along the beam propagation direction from the optimal CCD position for a fixed focal spot size of 50 *μ*m.

The transmission efficiency of the RZP was characterized at the BESSY II synchrotron facility. As displayed in Fig. 3(a), an average transmission efficiency of 11% is achieved in the spectral range from 250 to 600 eV, with the transmission efficiency increasing with photon energy. The observed photon energy-dependent transmission efficiency can be well reproduced by a simulation assuming a surface roughness of 0.2 nm rms, including a small oxidation^36^ and contamination layer of NiO_*x*_ and CO_*x*_ at an averaged atomic density of 4.6 g/cm^3^ by using the optical constants of C (43%), O (43%), and Ni (14%) and 2 nm thickness. We note that to provide higher RZP efficiency for higher energies, a groove depth of 9 nm was chosen with the RZP efficiency maximum shifted to 600 eV. Such a RZP behavior partially compensates a flux drop from the source at high energies.

**FIG. 3.**
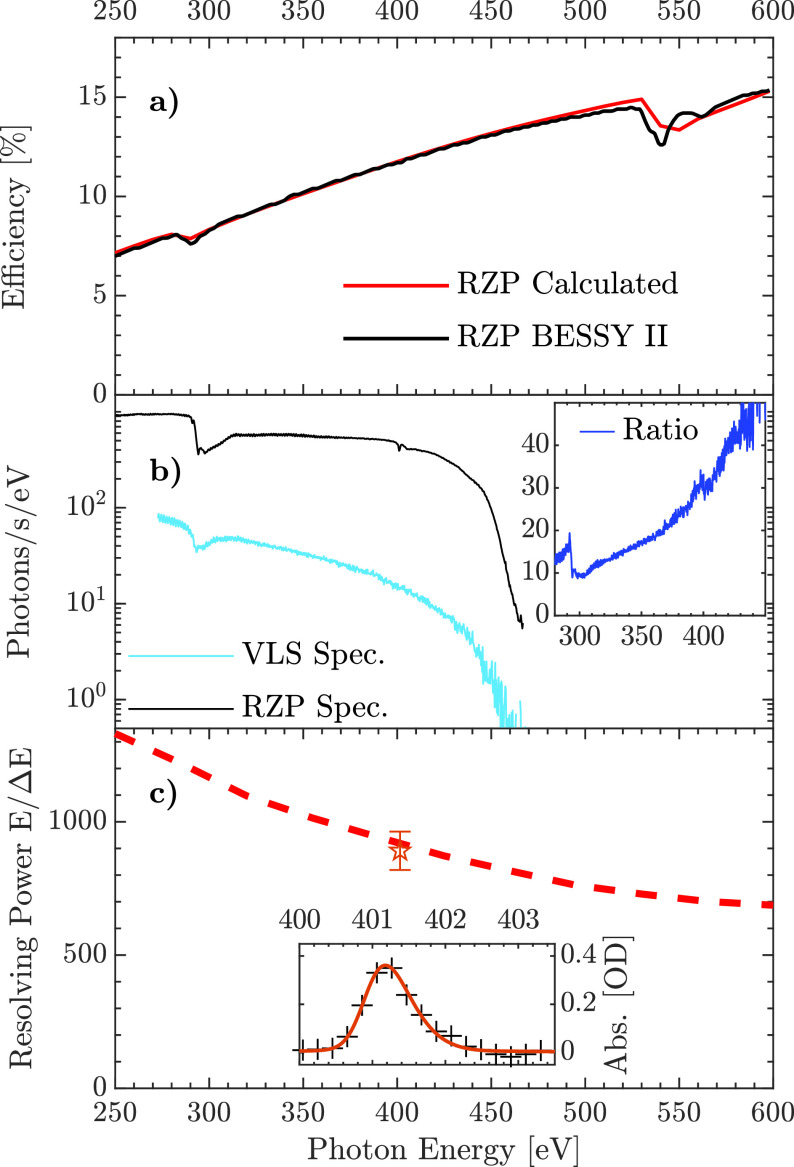
(a) RZP transmission efficiency, calculated including surface roughness and contamination (red, solid) and measured (black). (b) Soft x-ray high harmonic spectrum recorded previously with a VLS spectrometer^10^ (blue line) and with the new RZP spectrometer (black line). (c) Calculated (dashed lines) and measured (orange point) resolving power. The inset displays the absorption spectrum of the N(1s)- $\pi^{*}$ transition of gas-phase N_2_ recorded with the RZP spectrometer (black cross) and a fit (orange line, see text).

The RZP spectrometer performance was tested using our soft x-ray high-order harmonic source and compared with previous measurements done using a commercial VLS- and MCP-based spectrometer.^10^ Typical soft x-ray spectra resulting from HHG in helium by 1.8 *μ*m driving pulses are shown in Fig. 3(b) for both the VLS and the RZP spectrometers. In both cases, a broad spectrum extending from 270 eV to 450 eV is detected, with a large depletion of the HHG signal at the C K-edge and N K-edge, at a photon energy of 280 and 410 eV, respectively, both due to substantial contamination of the toroidal mirror M1. We note that the low-amplitude, high-frequency oscillations observe in the spectra correspond to the odd high-order harmonic structure. For the soft x-ray spectrum recorded with the VLS spectrometer, a 100-nm-thin aluminum filter was used to attenuate the infrared pulse co-propagating with the soft x-ray pulse. However, due to the relatively high-detection efficiency of the CCD camera used with the RZP spectrometer at a wavelength of 1.8 *μ*m, a 200-nm aluminum filter was required to fully attenuate the 1.8 *μ*m beam. Nevertheless, compared to the commercial VLS-based spectrometer, the total photon flux detected with the RZP spectrometer is 17 times higher, reaching 9.3 × 10^4^ photons/s. The throughput further increases as the photon energy increases [see inset in Fig. 3(b)], to finally reach a 30 times higher value at the N K-edge and a total of ≈400 photons/eV/s detected. This large improvement is due to the increased reflective efficiency of the RZP compared to the VLS grating and the quantum efficiency of the CCD detector, which is eight times higher with respect to the MCP/phosphor screen assembly used in the VLS spectrometer in our previous measurements.

The calculated energy resolving power of the RZP is shown in Fig. 3(c). To characterize the energy resolution of the spectrometer experimentally, the N K-edge absorption spectrum of gas-phase N_2_, $S_{N_{2}}$, was recorded [see inset in Fig. 3(c)] and then compared to a previously reported high-resolution spectrum *S_ref_* from Ohresser *et al.*^37^ convoluted with a Gaussian instrumental function using

$$S_{N_{2}}(E)=\frac{C}{\sqrt{2\pi \sigma^{2}}}\int S_{\mathrm{ref}}(E\prime )\cdot e^{\frac{-{\left(E-E^{\prime }\right)}^{2}}{2\sigma^{2}}}dE\prime ,$$ (1)

with *E* as the photon energy, *C* a scaling factor, and *σ* the width of the Gaussian distribution. The width of the Gaussian was adjusted by employing a least squares minimization, from which we could estimate the resolving power of the spectrometer by

$$\frac{E}{\Delta E}=\frac{E}{2\sqrt{2\;\text{ln}\;2}\sigma }.$$ (2)

The best fit was obtained for a resolving power of ≈890 ± 70 [orange line in inset of Fig. 3(c)], in very good agreement with the expected resolving power obtained from the simulations [see Fig. 3(c)].

To demonstrate the improved performance of the spectrometer in a soft x-ray absorption experiment, the soft x-ray absorption spectrum of a 750 mM aqueous solution of calcium nitrate (CaNO_3_) was measured. At the source point of the spectrometer unit, a liquid flatjet target was used formed by two 30-*μ*m-thick single laminar jets impinging onto each other under an 42° angle of incidence and mounted on motorized stages. The flow rate was adjusted to 1.8 ml/min in order to form a ≈3-*μ*m-thick and 300-*μ*m-wide liquid sheet^38,39^ at the focus of the soft x-ray pulse. A catcher with a 400-*μ*m-diameter nozzle was used to collect the liquid solution outside the vacuum chamber. The aforementioned aluminum filter mounted on a CF40 gate valve was used to isolate the x-ray spectrometer from the liquid jet, in order to avoid contamination of the x-ray optics by the large vapor pressure of water.

Figure 4(a) displays the absorption spectrum of CaNO_3_ obtained by averaging a total of 60 transmissions (I) and reference (i.e., without liquid sample) HHG spectra (I_0_), each integrated over a 12-s time window, by periodically moving the flatjet into and out of the HHG beam. To remove the contribution of the water solvent from the total absorption spectrum, the signal recorded below 340 eV was fitted to the known absorption of a pure water sample,^40,41^ using the thickness of the liquid flatjet as a fitting parameter [blue curve in Fig. 4(a)]. The best fit was achieved for a (2.9 ± 0.1)-*μ*m-thick liquid flatjet. After subtracting the water absorption, clear peaks are observed at 349.5 eV and 352.7 eV, corresponding to absorption at the Ca^2+^ L_2_- and L_3_-edges [see Fig. 4(b)]. In addition, strong absorption lines at 405.2 eV and 415 eV are observed, corresponding to the N(1s)- $\pi^{*}$ and N(1s)- $\sigma^{*}$ transitions in ${\text{NO}}_{3}^{-}$ anions, respectively.

**FIG. 4.**
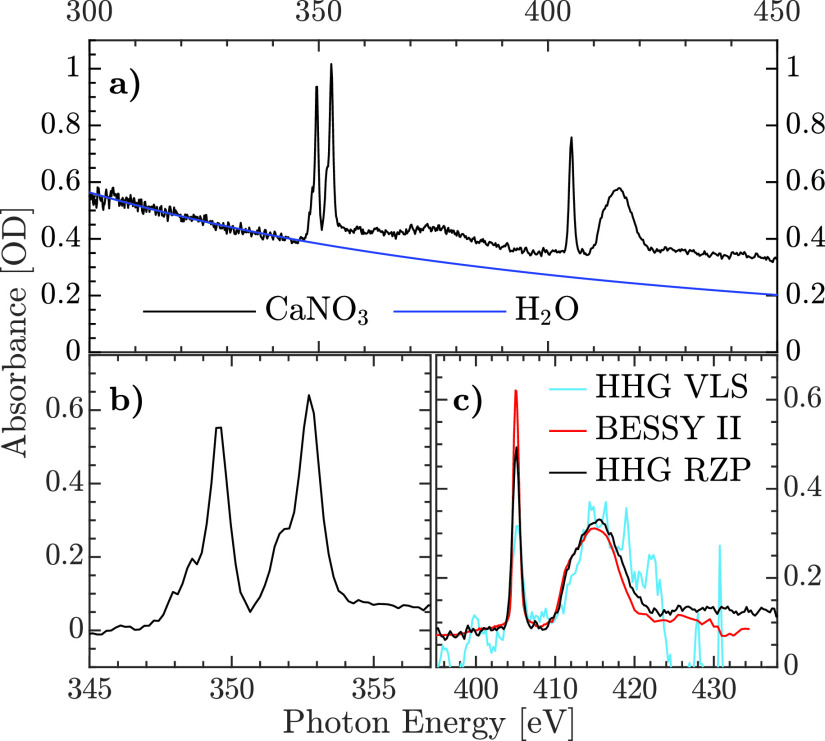
(a) Soft x-ray absorption spectrum of a 750 mM aqueous solution of calcium nitrate (black line), measured with an HHG source using the newly developed RZP spectrometer. The fitted absorption from the water solvent (blue line) is shown as well. The corresponding soft x-ray absorption spectra at the Ca L-edge and N K-edge obtained after subtracting the water contribution are displayed in panels (b) and (c), respectively. For comparison, the N K-edge absorption spectra of sodium nitrate (NaNO_3_) in solution recorded at the BESSY II synchrotron facility (red line) and in our previous experiments reported in Ref. 10 with a VLS spectrometer (blue line) are also shown.

In Fig. 4(c), the N K-edge absorption spectrum of CaNO_3_ measured using our broadband HHG source and the RZP spectrometer is compared with the N K-edge absorption spectrum of 750 mM NaNO_3_ in aqueous solution recorded in previous measurements using the VLS spectrometer.^10^ We note that in the previous measurements using the VLS spectrometer, the thickness of the liquid flatjet was estimated around 1.1 *μ*m, that is, a factor 2.6 times less than for our current results. To compare the two measurements, the absorption spectrum shown in Fig. 4(c) recorded in NaNO_3_ with the VLS spectrometer was therefore multiplied by this factor. In addition, we note that the difference of absorption cross sections between Ca and Na near the N K-edge has been accounted by adjusting an absorption offset below the N K-edge in order to have comparable absorption between the two measurements. In an aqueous solution, both molecules are characterized by the formation of ${\text{NO}}_{3}^{-}$ nitrate ions solvated by water molecules. The N K-edge absorption spectra of both, NaNO_3_ and CaNO_3_, in solution are therefore comparable, with strong absorption lines at 405.2 eV and 415 eV from the N(1s)- $\pi^{*}$ and N(1s)- $\sigma^{*}$ transitions occurring in the ${\text{NO}}_{3}^{-}$ anions. Comparing the two measurements, a total absorption of 757 ± 6 and 320 ± 60 mOD is measured at the N(1s)- $\pi^{*}$ absorption edge with the RZP and VLS spectrometer, respectively. The difference in absorption of the N(1s)- $\pi^{*}$ is assigned to the difference in resolving power between the two measurements. Importantly, a 30-fold improvement of the signal-to-noise ratio is achieved using the new spectrometer, which allows to reach an uncertainty below 1% of the absorbance. For completeness, a comparison with the N K-edge absorption spectrum of NaNO_3_ obtained at the UE52_SGM beamline at BESSY II recorded with a resolving power of 2800 is shown as well in Fig. 4(c). Clearly, the large increase in the detected photon count rate provided by the RZP spectrometer in combination with its high resolving power enables the recording of high-quality soft x-ray absorption spectra in the water window spectral range.

Even though we have obtained a large increase in the photon flux at the CCD detector, the accuracy that can been achieved with our current experimental setup is still limited by the shot noise and therefore follows the Poisson statistics. With a photon flux of 400 photons/eV/s at the N K-edge and considering a sample with a steady-state absorbance of 0.5 OD, 4 min of integration time will be required to reach an uncertainty of 1% relative to the static absorbance. In a pump-probe experiment composed of 50 time steps, less than 2 h of measurement is therefore required to detect a 1% change of absorption, which can be easily achieved by our source. Recently, static XAS at the N K-edge has been reported at the free electron laser FLASH facility.^42^ Using a transmission grating reference scheme, the high shot-to-shot fluctuations of SASE-FELs were compensated to the fundamental shot noise limited in XAS transmission mode measurements. With an average of 2700 photons/s detected in a 4 eV bandwidth, the achieved performance is similar to our table-top soft x-ray high harmonic setup.

The design and the characterization of a new soft x-ray spectrometer based on a reflective zone plate were presented. Compared to traditional VLS gratings, the RZP provides a higher transmission efficiency, which makes it ideally suited for soft x-ray absorption experiments with low-flux soft x-ray sources. In our current experiment, we detect a photon flux of 400 photons/eV/s, which is, to the best of our knowledge, the highest reported photon flux of detected photon at the N K-edge using a gas cell target for HHG. The spectrometer was used to measure high-resolution, high-quality, steady-state soft x-ray absorption spectra of molecules in solution with a femtosecond water window HHG source.

## Data Availability

The data that support the findings of this study are available from the corresponding author upon reasonable request.
